# Postbiotics and Skeletal Muscle Health: Molecular Mechanisms and Translational Perspectives

**DOI:** 10.3390/ijms27083470

**Published:** 2026-04-13

**Authors:** Matylda Korgiel, Maja Jakoniuk, Kacper Rak, Katarzyna Kler, Emil Paluch

**Affiliations:** 1 Faculty of Medicine, Wroclaw Medical University, Wybrzeze L. Pasteura 1, 50-367 Wroclaw, Poland; matylda.korgiel@student.umw.edu.pl (M.K.); maja.jakoniuk@student.umw.edu.pl (M.J.); kacper.rak@student.umw.edu.pl (K.R.); katarzyna.kler@student.umw.edu.pl (K.K.); 2 Department of Microbiology, Faculty of Medicine, Wroclaw Medical University, St. T. Chałubinskiego 4, 50-376 Wroclaw, Poland

**Keywords:** postbiotics, sarcopenia, gut–muscle axis, skeletal muscle, aging, muscle strength, muscle wasting, microbiota

## Abstract

Recent evidence implicates the gut microbiota in muscle physiology and function via the gut–muscle axis, which portrays bidirectional communication between microbial colonies, their metabolites and muscle tissue. Age-related muscle decline, including sarcopenia and muscle atrophy, has been associated with shifts in gut microbiota composition and lower levels of microbial metabolites, such as short-chain fatty acids (SCFAs), thereby expanding muscle health research toward microbiota-based therapies. Postbiotics, defined as preparations of inanimate microorganisms and/or their components, are gaining attention as a novel approach to combating muscle decline through modulation of microbiota–host communication, yet a comprehensive review of this topic is currently lacking. Preclinical studies demonstrate that postbiotics may exert anabolic effects while attenuating catabolism, inflammation, and cellular senescence, with associated improvements in grip strength, endurance capacity, and muscle morphology. Although clinical evidence remains limited, available studies indicate that postbiotics may have beneficial effects on muscle strength, endurance, and overall physical performance in humans. By synthesizing recent preclinical and clinical evidence, this review addresses an important gap in the literature, offering a comprehensive and mechanistically informed perspective on the potential role of postbiotics in modulating muscle health, particularly in the context of sarcopenia- and atrophy-associated muscle phenotypes.

## 1. Introduction

Sarcopenia is frequently associated with illnesses such as cancer, renal failure, liver disease, and metabolic disorders [1]. Advancing age, lack of physical exercise, a bad diet, malnutrition, smoking, diabetes, and excessive sleep are associated with a heightened risk of muscle disorders, including sarcopenia and muscle atrophy, both of which pose significant health risks in older adults [1,2,3]. According to the latest World Health Organization (WHO) data from 2025, the global population is aging at an unprecedented rate. By 2020, the global population aged over 60 had surpassed that of children under 5, and between 2015 and 2050, this age group is projected to double, increasing from 12% to 22% of the population [4].

Sarcopenia is a disease characterized by age-related loss of muscle mass, reduced muscle strength, and decreased functional capacity. Current estimates indicate that sarcopenia affects between 5% and 13% of individuals aged 60–70 years, despite the lack of uniform diagnostic standards [5]. With estimates that by the mid-21st century the global population of older adults will exceed 2 billion, the prevalence of sarcopenia will rise accordingly, becoming a new global health problem [6]. Calorie moderation and balanced dietary patterns are considered effective methods for combating progressive muscular changes, while standard management also emphasizes strength training to improve muscle function [2,7]. Muscle atrophy refers to reduction of muscle volume, specifically reduction in myofiber size, arising from factors such as aging, chronic illnesses or insufficient muscle loading [8]. The prevalence of muscle wasting across chronic diseases is estimated at 24.2–40.4%, and gradual atrophy has been associated with reduced daily activity, thus contributing to disease progression and higher mortality [9].

In recent years, the relationship between the gut microbiota and muscle metabolic pathways has attracted increasing scientific interest [10]. The gut microbiota comprises a vast community of microorganisms residing in the gastrointestinal tract, numbering more than 10^14^ cells. It is dominated by four major phyla: Firmicutes, Bacteroidetes, Proteobacteria, and Actinobacteria [11]. Advances in sequencing technologies have revealed over 9.9 million microbial genes in the human gut and more than 1000 predominantly anaerobic bacterial species, with composition shifting across the lifespan [12,13]. This diverse ecosystem plays a comprehensive role throughout the body, from basic digestion and absorption to cooperation with the immune system [14]. The microbiota and host interact bidirectionally from the moment they are born. Throughout this connection called cross-talk, the gut immune system continually checks the environment, playing an important role in distinguishing the symbiotic flora from dangerous infections [15]. The human form is a holobiont, a superorganism composed of human cells and trillions of microorganisms. These bacteria, which make up the majority of our genetic material, serve as a protective barrier, ensuring the organism’s interaction with its surroundings [16]. Genes involved in the digestion of proteins, lipids, carbohydrates, and vitamins have been found to be reduced in sarcopenia; this may be directly related to the altered microbiota composition [17]. Gut microbiota not only supports intestinal homeostasis but also facilitates two-way communication with distant organs, including skeletal muscle, via its metabolic products, helping to maintain overall physiological balance. This framework has led to the development of the “gut-muscle axis” concept [18]. Aging and other environmental factors can perturb gut microbiome diversity, often leading to dysbiosis—defined as alterations in the composition and functionality of the microbiota, typically involving the loss of symbiotic strains in favour of the overgrowth of harmful microbes [19,20,21]. Pathological overgrowth of Proteobacteria (particularly *Escherichia*, *Klebsiella*, and *Bilophila*), as well as *Clostridium*, *Enterococcus*, and *Lactobacillus* at the expense of Bacteroidetes, causes elevated lipopolysaccharide (LPS) levels and intestinal leakage, which, via antigen translocation, fuels chronic inflammation that underpins multimorbidity [22]. LPS is produced by the microbiota and enters the circulation by direct diffusion, causing endotoxemia—elevated amounts of LPS in the bloodstream—particularly in conditions characterized by increased intestinal permeability, including high-fat dietary intake, obesity, type 2 diabetes, and non-alcoholic fatty liver disease. Both acute infections and chronic illnesses involve LPS in their pathogenesis, which aids in the generation of cytokines and acute-phase proteins [23]. Studies have shown that proper functioning of the gut-muscle axis, vast biodiversity of the microbiome, and the number of bacteria synthesizing short chain fatty acids (SCFAs) can reduce the consequences of sarcopenia and muscle mass loss, suggesting that muscle phenotypes may depend on gut health [7,24,25,26].

Interventions in the microbiome, such as supplementation of probiotics, prebiotics and postbiotics, are considered a viable method for regulating microbiota balance and improving intestinal homeostasis [27]. Bacterial depletion, faecal microbiota transplantation (FMT), and nutrition are further microbiome interventions that have been demonstrated to affect muscle phenotypes [22,28]. The potential benefits of using prebiotics, probiotics, and postbiotics are an active area of research and hold therapeutic potential for numerous systemic diseases believed to be linked to dysbiosis [29].

The use of the term “postbiotic” has increased significantly in recent years. After many attempts, in 2021, the International Scientific Association for Probiotics and Prebiotics (ISAPP) proposed a definition of postbiotics as “a preparation of inanimate microorganisms and/or their components that confers a health benefit on the host” [30]. Postbiotics are generated through fermentation and bacterial processing and may comprise not only microbial cells but also the bioactive components of the fermentation matrix, including SCFAs, organic acids, oligosaccharides, peptides, and vitamins [31]. Research indicates that these products may help strengthen the body’s defences and enhance the integrity of the intestinal barrier [20,31]. It should be noted that some authors distinguish paraprobiotics as a separate category, referring to inactivated microbial cells or their structural components, particularly cell wall–associated molecules [32]. However, consistent with current ISAPP terminology, these structural bacterial components are discussed in this review as postbiotics, together with soluble metabolites and other secreted bioactive compounds.

In the following work, we explore the potential of postbiotics to improve muscle health, situating this discussion within the broader concept of the gut–muscle axis and examining the molecular pathways through which postbiotics may exert their effects. As current literature lacks a comprehensive overview dedicated specifically to postbiotics in the context of muscle disease, this review seeks to address this gap by synthesizing evidence from preclinical models alongside the limited number of available clinical studies completed to date, while considering future directions and acknowledging current limitations.

## 2. Role of Gut Microbiota in Muscle Function and Metabolism

### 2.1. The Gut-Muscle Axis

The gut–muscle axis describes the bidirectional communication between gut microbiota and skeletal muscle, integrating metabolic, endocrine, and immune pathways through which microbial communities influence muscle physiology [12,33]. As a metabolic organ, the gut microbiota converts indigestible dietary components, mainly dietary fibres and resistant starches, and, to a lesser degree, proteins, into bioactive metabolites that take part in regulating inflammation, immunity, endocrine signalling, metabolic pathways, and insulin sensitivity—key processes shaping muscle mass and function. Communication along the axis is mediated by hormones, cytokines and extracellular vesicles (EVs), with both the gut and muscle acting as signalling hubs [12,33,34]. Crosstalk between skeletal muscle and the gut microbiome reflects a dynamic feedback loop in which microbial signals shape muscle metabolism and regenerative capacity, while muscle-derived factors and metabolic demands, in turn, influence microbial composition and function, jointly determining musculoskeletal health across the lifespan [34].

**Figure 1:**
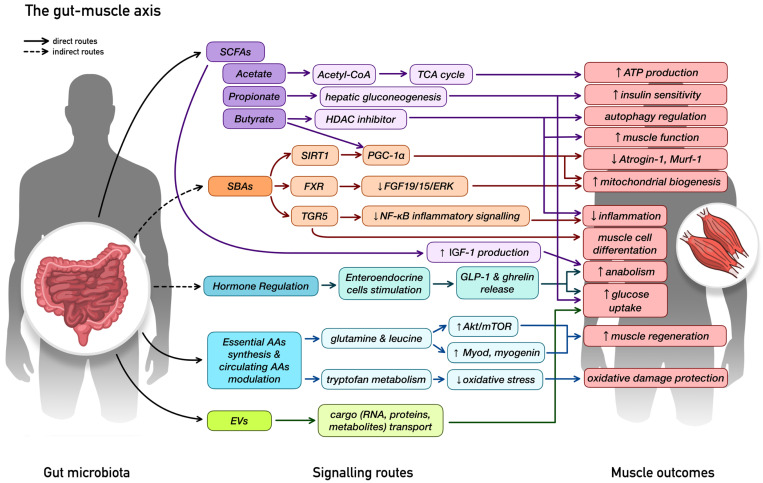
The gut microbiota influences skeletal muscle through direct (solid black lines) and indirect (dashed black lines) signalling routes, classified according to microbial origin. Direct routes include microbiota-derived mediators such as SCFAs, AAs, and EVs, which can also potentially engage in secondary host-mediated mechanisms, while indirect routes reflect microbiota-mediated modulation of host factors, including SBAs, generated through microbial conversion of PBAs, and hormone secretion. SCFAs, such as acetate, propionate, and butyrate, act as metabolic and immune signals, engaging in energy production via the TCA cycle, enhancing insulin sensitivity and glucose uptake, while also inhibiting HDACs to maintain immune balance. Butyrate also activates the SIRT1/PGC-α pathway, which collectively improves muscle function, regulates autophagy, and suppresses the expression of catabolic E3-ubiquitin ligases Atrogin-1 and Murf-1. Microbiota-derived AAs and their metabolites modulate anabolic signalling, particularly through Akt/mTOR pathways and myogenic regulatory factors (MyoD, myogenin), supporting muscle protein synthesis and regeneration. SBAs signal through the FXR to modulate the FGF19/15/ERK pathway and through the TGR5 receptor to inhibit NF-κB-driven inflammatory signalling, while also engaging SIRT1/PGC-1α pathways to promote mitochondrial efficiency and muscle cell differentiation. In parallel, the gut microbiota modulates hormone secretion, including GLP-1, ghrelin, and IGF-1, which collectively support muscle glucose utilisation, mitochondrial activity, and maintenance of muscle mass. Notably, SCFAs may also contribute to hormone-mediated signalling. Signalling routes are colour-coded according to the main mediators (SCFAs, AAs, EVs, SBAs, hormones) and their associated cascades; muscle-related outcomes are highlighted in pink. Abbreviations: SCFAs—short-chain fatty acids; SBAs—secondary bile acids; AAs—amino acids; EVs—extracellular vesicles; TCA—tricarboxylic acid; ATP—adenosine triphosphate; HDAC—histone deacetylase; SIRT1—sirtuin 1; PGC-1α—peroxisome proliferator-activated receptor gamma coactivator 1-alpha; FXR—farnesoid X receptor; FGF19/15—fibroblast growth factor 19/15; ERK—extracellular signal-regulated kinase; TGR5—Takeda G protein-coupled receptor 5; NF-κB—nuclear factor kappa-light-chain-enhancer of activated B cells; GLP-1—glucagon-like peptide-1; Akt—protein kinase B; mTOR—mechanistic target of rapamycin; IGF-1—insulin-like growth factor 1.

Figure 1 [11,12,18,33,34,35,36,37] provides a schematic representation of the main signalling routes of the gut-muscle axis, encompassing SCFAs derived from dietary fibre fermentation, microbiota-generated amino acids (AAs) and secondary bile acids (SBAs), microbial EVs, and hormone secretion modulation.

Beyond producing key metabolites, the gut microbiome contributes essential micronutrients that directly support muscle maintenance and resilience. For example, microbial synthesis of vitamins—including B vitamins and vitamin K—supports energy pathways and protects against muscle atrophy, particularly during inflammation [34]. The gut microbiome also helps maintain NAD^+^ homeostasis, and because NAD^+^ is essential for the tricarboxylic acid (TCA) cycle and oxidative phosphorylation, this function is critical for preserving mitochondrial efficiency. Notably, low NAD^+^ levels are closely linked to sarcopenia [36]. Together, these mechanisms highlight the relevance of gut microbes as important regulators of muscle mass and function.

Evidence shows that specific gut bacteria positively influence muscle strength. Higher muscle performance is associated with *Lactobacillus johnsonii*, *Limosilactobacillus reuteri*, and *Turicibacter sanguinis*, with *L. johnsonii* and *L. reuteri* shown to enhance strength in mice, possibly via upregulation of IGF-1 and follistatin [38]. *Eisenbergiella massiliensis* and *Anaeroplasma abactoclasticum* are also enriched in stronger-muscle profiles [39]. The impact of specific bacteria on skeletal muscle mass, strength, and metabolism is summarized in Table 1.

Accumulating evidence shows that muscle metabolism and function are strongly influenced by gut microbial composition and abundance, and that disturbances in these interactions contribute to age-related muscle deterioration [35,42]. Providing mechanistic evidence for this relationship, germ-free mice without gut microbiota exhibit muscle atrophy, lower IGF expression, and downregulation of genes involved in muscle growth and mitochondrial function. Notably, transplanting faecal microbiota from pathogen-free mice restores muscle mass, reduces atrophy markers, and improves muscle oxidative metabolism [43]. Consequently, targeting the gut microbiota has emerged as a promising strategy for the prevention or management of muscle-related conditions, including sarcopenia and muscular dystrophy [12,34,35].

### 2.2. Age-Related Alterations of the Gut Microbiota and Muscle Health

Age-related changes in the gut microbiota may contribute to anabolic resistance by promoting inflammation, oxidative stress, insulin resistance, increased intestinal permeability and metabolic endotoxemia. These alterations can disrupt amino acid homeostasis, reduce microbial production of key metabolites and affect pathways crucial for muscle protein synthesis, including IGF-1 and PI3K/Akt signalling. Gut dysbiosis may also impair nutrient absorption and modulate endocrine and immune responses, further accelerating muscle loss [44].

#### 2.2.1. Age-Associated Microbiota Dysbiosis and Muscle Function

Individuals with reduced muscle function or sarcopenia tend to exhibit lower gut microbial diversity and a decreased abundance of beneficial butyrate-producing bacteria [45]. A systematic review by Ren et al. further supports the association between gut microbiota composition and muscle health in older adults, with sarcopenia linked to a less resilient, more dysbiotic ecosystem associated with declining muscle mass and function [46].

Numerous studies confirm that certain microbial strains may be associated with muscle-related outcomes. Frail or sarcopenic individuals exhibit depletion of beneficial taxa and higher abundance of *Eggerthella*, *Bacteroides*/*Prevotella*, *Lactobacillus*/*Enterococcus*, *Oscillospira*, and *Ruminococcus*, reflecting a dysbiotic gut linked to impaired muscle function [44]. High prevalence of bacterial species such as *Prevotella*, *Agathobacter*, and *Alloprevotella* was linked to greater muscle strength and healthier muscle profiles [46], while increased levels of *Prevotella* and *Barnesiella* (including *Barnesiella intestinihominis*) also correlated with superior muscle strength in older adults [47]. In contrast, elevated levels of *Escherichia-Shigella*, *Eggerthella*, and *Collinsella aerofaciens* were more common in individuals with sarcopenia, pointing to a potential detrimental role in muscle function [46]. Supporting these findings, 16S rRNA sequencing showed increased levels of *Lactobacillus* alongside decreased abundance of the genera *Fusicantenibacter*, *Eubacterium*, *Lachnospira*, *Lachnoclostridium* and *Roseburia* in sarcopenic patients compared with healthy controls [48].

#### 2.2.2. Loss of Gut Homeostasis and Intestinal Barrier Integrity

The gut microbiota supports metabolic homeostasis and immune function by maintaining intestinal barrier integrity [35], which enables nutrient absorption while preventing translocation of pro-inflammatory components like LPS [34]. Loss of gut homeostasis increases the levels of Gram-negative, LPS-containing bacteria—such as *Escherichia/Shigella*, *Klebsiella*, and *Citrobacter*—which drive systemic inflammation [11]. Disruption of the gut barrier allows LPS and other microbial products to enter circulation, activating inflammatory pathways (NF-κB, MAPKs) that upregulate atrophy-related genes such as Atrogin-1 and MuRF-1 and promote proinflammatory cytokines (e.g., IL-6, TNF-α), thereby accelerating muscle wasting and contributing to sarcopenia progression [35,36,42]. Furthermore, the lack of gut microbiota or antibiotic-induced dysbiosis alters muscle atrophy markers (FoxO, Atrogin-1, Murf-1, MyoD) and reduces expression of genes linked to muscle growth (ERK1/2, p90RSK, RPS6) and mitochondrial function (PGC-1α, AMPK) [37].

#### 2.2.3. Functional Consequences of Microbiota Alterations: SCFAs and Bile Acid Signaling

SCFA levels generally decline with age, partly due to gut dysbiosis, including reduced *Lachnospiraceae* [33]. Sarcopenic individuals showed distinct species-level shifts in their faecal microbiota, including marked reductions in the SCFA-producing species *Faecalibacterium prausnitzii* and *Roseburia inulinivorans*, as well as a decline in *Alistipes shahii* [40]. Lower extracellular SCFAs may impair muscle function via their receptors GPR43 and GPR41, with GPR43 being the primary mediator [33]. Because SCFAs support glucose uptake through GPR43/41 receptors and enhance mitochondrial biogenesis via SIRT1 activation, reduced SCFA levels can lead to insulin resistance and mitochondrial dysfunction [42]. Reduced butyrate—critical for enterocyte energy—leads to glucose compensation, lowering serum glucose, altering insulin sensitivity, and increasing hepatic gluconeogenesis. Dysbiosis can also decrease skeletal muscle glucose availability, reducing glycogen stores, which impairs aerobic energy metabolism, muscle strength, and overall bioenergetic function [11]. A decline in muscle butyrate levels may contribute to the loss and functional impairment of aging satellite cells, with reduced butyrate and gut microbial dysbiosis likely driving the decrease in satellite cell abundance and regenerative capacity in aging muscle [49].

Gut dysbiosis may drive sarcopenia via the bile acid–FXR pathway. It has been proposed that loss of bile salt hydrolase (BSH)-producing bacteria reduces secondary bile acid formation, leading to accumulation of tauro-β-muricholic acid (TβMCA), a Farnesoid X Receptor (FXR) antagonist. Elevated TβMCA may suppress FXR–FGF15/19 signalling, resulting in attenuated FGFR4–KLB signalling in skeletal muscle and reduced activation of downstream ERK1/2, p90RSK, and RPS6, which impairs muscle protein synthesis and contributes to muscle atrophy, reduced muscle mass, and diminished strength [33,36]. This proposed mechanism is largely based on the work of Qie et al., who reported that antibiotic-treated mice exhibited a bile acid profile dominated by the FXR antagonist TβMCA, accompanied by a marked reduction in bile salt hydrolase (BSH)–active gut microbes, including *Lactobacillus* and *Bifidobacterium*, and an associated decrease in skeletal muscle mass [41].

**Figure 2:**
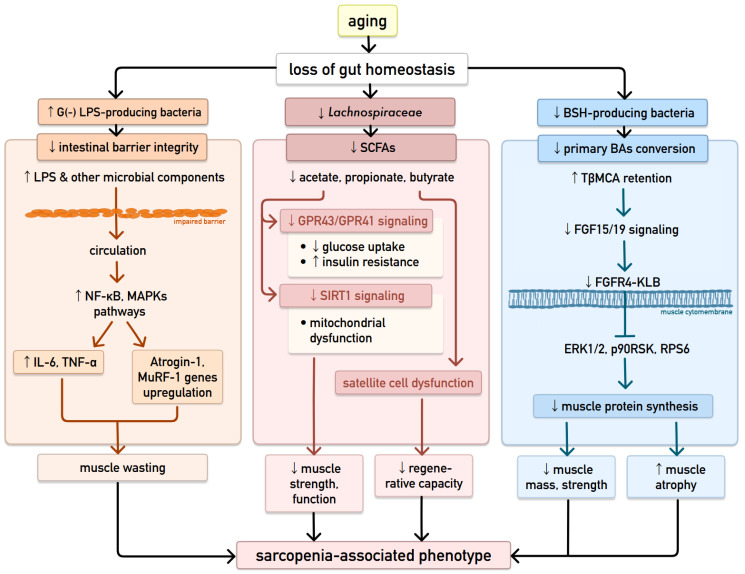
Aging-associated decrease in intestinal barrier integrity and loss of gut homeostasis drive pathways leading to muscle deterioration and a sarcopenia-associated phenotype [11,33,35,36,37,41,42,49]. Abbreviations: LPS—lipopolysaccharides; NF-κB—nuclear factor kappa B; MAPKs—mitogen-activated protein kinases; IL-6—interleukin 6; TNF-α—tumour necrosis factor alpha; SCFAs—short-chain fatty acids; GPR—G protein–coupled receptor(s); SIRT1—sirtuin 1; BSH—bile salt hydrolase; BAs—bile acids; TβMCA—tauro-β-muricholic acid; FGF15/19—fibroblast growth factor 15/19; FGFR4–KLB—fibroblast growth factor receptor 4–β-Klotho complex; ERK1/2—extracellular signal-regulated kinase 1/2; p90RSK—p90 ribosomal S6 kinase; RPS6—ribosomal protein S6.

Figure 2 summarizes how aging-associated loss of gut homeostasis and impaired intestinal barrier integrity, which result in altered microbial metabolite signalling, converge on pathways driving muscle deterioration and a sarcopenia-associated phenotype [11,33,35,36,37,41,42,49].

## 3. Postbiotics Overview

As defined by ISAPP, postbiotics refer to preparations of inanimate microorganisms and/or their components that confer a health benefit to the host. The term combines biotic, meaning “derived from living organisms,” with the prefix post, meaning “after,” reflecting the concept of non-living microbial components that exert biological effects [30].

Postbiotics comprise microbial-derived bioactive molecules classified into two main groups: bacterial structural components and bacterial metabolites [50], as summarized in Table 2.

The main categories of postbiotics comprise cell-free supernatants rich in organic acids, bacteriocins, and proteins; SCFAs; exopolysaccharides; enzymes; vitamins; neurotransmitters; and extracellular vesicles [51]. By molecular weight, they are divided into high- and low-molecular-weight postbiotics [52]. Structurally, they include peptides, teichoic acids, and plasmalogens. In terms of composition, postbiotics comprise carbohydrates (teichoic acids, galactose-rich polysaccharides), proteins (p40, p75 molecule, lactocepin), lipids (including SCFAs, lactate, and dimethyl acetyl-derived plasmalogens), vitamins (e.g., B-group vitamins), and other complex molecules (such as muropeptides) [52,53].

### 3.1. Stability and Safety of Postbiotics

Postbiotics are attracting increasing interest due to their superior stability compared with probiotics during production and storage. Unlike live microorganisms, which are sensitive to environmental factors and lose viability over time, inactivated microbial products remain stable at room temperature and retain consistent functional properties, enabling predictable dosing and simpler storage requirements [30,54]. In contrast to probiotics, whose viable cell counts decline unpredictably and may complicate formulation consistency and clinical evaluation, postbiotics maintain a fixed composition from manufacture to end use [30].

Additionally, postbiotics offer a favourable safety profile because they contain non-replicating microorganisms, eliminating the risk of bacteraemia or fungemia and reducing concerns related to genetic modification or horizontal gene transfer [30,55]. Their use also avoids the potential transfer of antibiotic resistance genes and is particularly suitable for vulnerable populations, such as children, with developing immune systems and intestinal barriers [56]. Importantly, postbiotics can exert direct beneficial effects on the host independently of probiotic viability or prebiotic activity [52].

### 3.2. Molecular Mechanisms of Postbiotic Action

ISAPP experts identified five key mechanisms of action of postbiotics: (1) influencing the composition and activity of the resident microbiota, (2) strengthening epithelial barrier function, (3) regulating both local and systemic immune responses, (4) affecting metabolic processes, and (5) mediating systemic communication through the nervous system [30].

**Figure 3:**
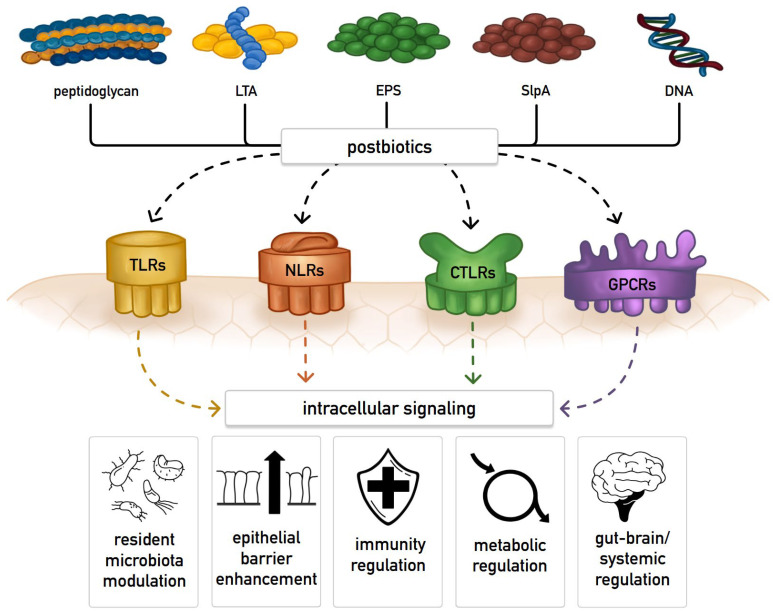
Proposed postbiotic–host receptor interactions underlying consensus mechanisms of action [30,32,57,58]. Postbiotics are thought to exert their effects by interacting with host–cell receptors. As bacterial metabolites and structural components—such as peptidoglycan, lipoteichoic acid (LTA), exopolysaccharides (EPS), S-layer protein A (SlpA), and DNA—they engage pattern-recognition receptors (PRRs) and trigger beneficial signalling pathways. Key PRRs include Toll-like receptors (TLRs) that detect microbe-associated molecular patterns (MAMPs). Various *Lactobacillus*-derived postbiotic components, such as EPS and surface layer proteins, have been shown to activate these receptors to modulate MAPK and NF-κB pathways, collectively reducing inflammation in intestinal epithelial cells. Nucleotide-Binding Oligomerization Domain-Like Receptors (NLRs) sense peptidoglycan fragments and activate anti-inflammatory and antibacterial responses. C-Type Lectin-Like Receptors (CTLRs) bind microbial glycans and modulate immune activity, while G-Protein-Coupled Receptors (GPCRs) respond to microbial SCFAs to regulate immunity, metabolism, and epithelial function.

Figure 3 illustrates how postbiotic components interact with host pattern-recognition receptors to activate key signalling pathways that underlie the consensus mechanisms of postbiotic action [30,32,57,58].

The five principal mechanisms through which postbiotics influence host physiology, together with representative components, targets, and functional outcomes related to muscle health, are summarized in Table 3 [32,50,54,57,59,60,61,62,63,64,65,66,67,68,69]. Most mechanisms listed are derived from preclinical models and represent plausible pathways for muscle health modulation.

## 4. Postbiotics and Skeletal Muscle: Evidence from Preclinical Models

Animal models enable precise assessment of postbiotic effects on muscle fibre structure and signalling pathways in the context of muscle atrophy–related phenotypes. Such mechanistic detail is difficult to achieve in human studies, as muscle biopsies are invasive and ethically constrained. The following section synthesizes the current state of knowledge regarding the impact of postbiotics on morphological and functional parameters of skeletal muscle in experimental models.

### 4.1. Functional and Phenotypic Outcomes of Postbiotic Supplementation

Inhibition of muscle atrophy, enhancement of muscle strength and performance, and prevention of muscle function impairment are primary endpoints in the preclinical evaluation of therapies aimed at combating atrophy, a condition characterized by a progressive decline in skeletal muscle mass and functional capacity, driven intracellularly by a disruption of homeostasis between anabolic and catabolic signaling cascades [10,70,71,72,73,74,75]. The collected data indicate that supplementation with postbiotics leads to measurable phenotypic benefits, which are evident at both the macroscopic level—such as increased muscle weight—and the functional level, as seen in performance tests [71,72,73].

Dexamethasone (DEX) is a synthetic glucocorticoid commonly used in preclinical studies to induce myofibre atrophy, as it inhibits muscle protein synthesis and promotes proteolysis [76,77]. Most studies examining postbiotics in the context of muscle atrophy indicate protective or myogenic effects on muscle mass, strength, and performance [70,71,72,73,74,75]. Park et al. investigated the effects of DuoX—a combination of postbiotic beLP1, previously suggested to exert anti-sarcopenic activity, and *C. intybus* L. root extract containing inulin, which has known anti-inflammatory properties—on the grip strength in rats with DEX-induced myofiber atrophy. DEX administration resulted in a marked reduction in grip strength when compared to the controls. Notably, oral supplementation with postbiotic DuoX attenuated this decline [70]. A similar result, showing improved grip strength, was demonstrated in a study on the effect of KL-Biome (a postbiotic formulation of *Lactiplantibacillus plantarum* KM2) on the muscles of DEX-treated rats [72]. A study by Han et al. used a different method to induce muscle atrophy in mice, which involved immobilization of the hindlimbs. The researchers tested the anti-sarcopenic effects of Whey + DH5 + KEE postbiotics (WDK) produced from a polyphenol-rich melon peel extract (*Cucumis melo* L. var. *makuwa*, KEE) and whey fermented with *Lentilactobacillus kefiri* DH5 (DH5) and also reported significant improvements in the grip strength [73]. Further supporting this, a study in seven-month-old pre-sarcopenic senescent accelerated mouse prone 8 (SAMP8) mice found that supplementation with an SCFA cocktail over three months led to significant improvements in muscle mass and grip strength compared with control mice receiving sodium water [74]. The hindlimb immobilization model was also used in a study conducted by Jeong et al. Their study confirmed that the postbiotic product, Whey + DH5 + GSE (WDG), obtained from the bioconversion of grape seed flour extract (GSE) and branched-chain amino acid (BCAA)-rich whey using DH5, significantly improved grip strength [75].

Additionally, several studies have demonstrated that postbiotics improve exercise capacity [71,72,74]. Jeon et al. used a forced swimming test to assess fatigue and exercise-induced endurance. Conclusions from the test were drawn based on differences in blood levels of enzymes such as ALT, AST, ALP, LDH, CK, and lactate [71]. Elevated AST, ALT, and ALP levels typically indicate liver injury and inflammation, but may also increase after intense physical exercise [78]. Similarly, LDH and CK levels can rise with intense aerobic exercise, while lactate accumulation in fatigued muscles reflects enhanced anaerobic glycolysis [79,80]. In the cited study, mice supplemented with *Lactobacillus curvatus* HY7602 fermented deer antler (FA) exhibited reduced levels of the aforementioned enzymes and lactate, suggesting a potential beneficial effect on exercise endurance [71]. Consistent with these findings, treadmill-based endurance testing revealed superior anti-fatigue performance in aged sarcopenic mice receiving an SCFA cocktail [74]. Similarly, treadmill exercise testing showed that KL-Biome administration restored moving distance and speed in DEX-treated mice to levels comparable with the normal control group, indicating improved exercise capacity and resistance to fatigue [72].

These findings support the potential benefits of postbiotic action on muscle function, including improvements in grip strength and endurance capacity.

### 4.2. Mechanistic Basis of Postbiotic-Induced Muscle Adaptations

Improved physical performance in supplemented animals likely reflects the synergistic interplay between anabolic and regenerative processes at the tissue level. To better understand the molecular basis underlying the observed functional improvements, which suggest that postbiotics actively foster an anabolic environment in muscle tissue, several studies subsequently analysed the impact of postbiotics on protein anabolic pathways [71,72,73,74,75].

Anabolic Support

Key to the anabolic processes is the mTOR kinase pathway, which regulates protein synthesis [81]. Research indicates that supplementation with various postbiotics activates the mTORC1 signalling cascade, promoting the phosphorylation of essential effector proteins such as p70S6K, eIF4E, ULK-1, and 4E-BP1. This corroborates that, even under conditions of metabolic stress, the translation process is stimulated, leading to the synthesis of new contractile proteins [82]. In vitro and in vivo studies have shown that SCFAs activate mTOR signalling, thereby promoting myocyte growth [74]. Similar conclusions were drawn in the study on the postbiotic KL-Biome, during which mTOR phosphorylation, reduced by DEX, significantly increased after KL-Biome treatment [72]. The shift towards increased protein anabolism has also been documented in muscle cell–based systems, as an increase in myotube diameter in C2C12 cells, which reflects enhanced muscle fibre growth [70,72,73,74].

Postbiotics exert effects beyond just mTOR-mediated muscle hypertrophy. Supplementation may also affect myogenic regulatory factors (MRFs), particularly MyoD, Myf5, and myogenin [71,73,74,75,83]. The transcription factors mentioned have the ability to convert different types of differentiated cells into muscle cells through the process of myogenesis [84].

The interaction between increased protein synthesis via the mTOR pathway and stimulation of transcription factor-dependent myogenesis results in a phenotypic change characterized by increased muscle mass and enhanced physical performance. In contrast, a study by Liu et al. showed that myogenic gene expression was similar to that of the control group. However, there was a significant reduction in the expression of genes responsible for muscle atrophy [74].

Additionally, an in vitro study using C2C12 myoblasts has shown that butyrate stimulates myoblast proliferation by promoting G1/S cell cycle transition via activation of the ERK/MAPK pathway. This indicates that butyrate may support muscle regeneration and counteract age-related muscle loss by enhancing myoblast proliferation and potentially improving muscle maintenance [67].

In conclusion, postbiotics facilitate muscle anabolism by activating the mTOR pathway and myogenic regulatory factors. Moreover, butyrate specifically aids regeneration and combats atrophy through the ERK/MAPK signalling cascade.

Anti-catabolic Effects

Dysregulation of protein synthesis and degradation, which promotes catabolic processes in muscle tissue, leads to muscle mass loss in sarcopenia. The ubiquitin-proteasome system (UPS) is the primary mechanism of muscle protein proteolysis. The main transcription factors responsible for atrophy in aging are FOXO factors, which stimulate the expression of atrophic genes and the formation of the ubiquitin ligases Atrogin-1 and MuRF1 [85]. KL-Biome supplementation effectively inhibited FoxO3a expression, which, in turn, reduced MuRF1 and atrogin-1 expression, suggesting that KL-Biome improved DEX-induced muscle atrophy [72]. Similar conclusions indicating the possible effectiveness of postbiotics in the treatment of muscle atrophy due to the inhibition of catabolism were shown by studies on SCFAs [74], DuoX [70], WDG [75], postbiotic *Lactiplantibacillus plantarum* HY7717 [83], and postbiotic EVs [86].

Anti-inflammatory Actions

Additionally, preclinical cell-based models provide evidence supporting the anti-inflammatory properties of postbiotics. Chronic low-grade inflammation is a key contributor to sarcopenia, as it activates proteolytic pathways that promote muscle protein degradation [85]. Available evidence suggests that postbiotics can mitigate inflammation-induced muscle catabolism by reducing the expression of pro-inflammatory cytokines, including interleukin-6 (IL-6), tumour necrosis factor-α (TNF-α), and interleukin-1β (IL-1β), in skeletal muscle [70,71,83]. In this context, postbiotic-mediated modulation of inflammatory signalling represents a mechanistic link between the gut microbiota and the preservation of muscle mass and function.

Anti-aging Effects

In parallel with these muscle-directed effects, postbiotics have also been investigated in models of age-related cellular stress, which is closely intertwined with chronic inflammation and sarcopenia progression. Age-associated alterations in gut microbiota composition and metabolic capacity contribute to musculoskeletal dysfunction and sarcopenia [10,87]. Work by Siddharth et al. underscores the importance of microbial metabolic pathways—particularly those involved in lipid, carbohydrate, and vitamin metabolism—in the aging process [10]. Supporting this concept, an H_2_O_2_-induced aging model in 3T3-L1 preadipocytes demonstrated that a postbiotic secretory metabolite from *Lactobacillus fermentum* attenuated PI3K/Akt/mTOR pathway phosphorylation and reduced multiple senescence-associated markers, including p53, p21^WAF1, SA-β-gal, p38 MAPK, iNOS, COX-2, NF-κB, and reactive oxygen species (ROS) [87]. Although not a muscle-specific model, these findings provide mechanistic support for the anti-aging and anti-inflammatory potential of postbiotics, which may be relevant to the modulation of pathways associated with muscle decline.

**Figure 4:**
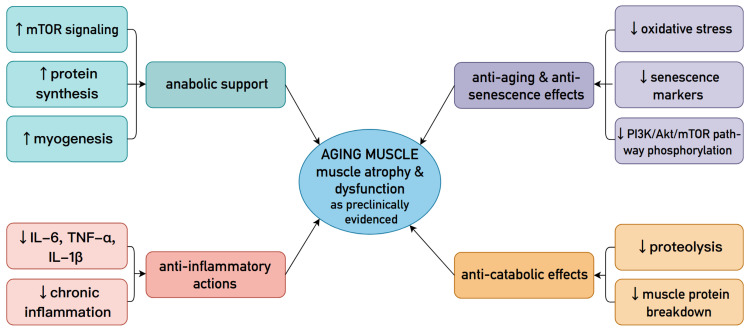
Summary of Preclinical Mechanisms Through Which Postbiotics Modulate Age-Related Muscle Atrophy [70,71,72,73,74,75,83,87]. Abbreviations: mTOR—mechanistic target of rapamycin; IL-6—interleukin 6; TNF-α—tumour necrosis factor alpha; IL-1β—interleukin 1 beta; PI3K/Akt/mTOR—phosphoinositide 3-kinase/protein kinase B (Akt)/mechanistic target of rapamycin.

Collectively, evidence from preclinical models and preliminary studies indicates that postbiotics can influence age-related muscle atrophy by modulating anabolic, catabolic, inflammatory, and senescence-associated pathways. To integrate the functional and mechanistic findings discussed in Section 4.1 and Section 4.2, Figure 4 summarizes the principal preclinical pathways through which postbiotics may modulate age-related muscle atrophy [70,71,72,73,74,75,83,87].

## 5. Postbiotics and Muscle-Related Outcomes: Evidence from Clinical Studies

Although preclinical studies suggest a potential therapeutic role for postbiotic supplementation in muscle atrophy and sarcopenia, human evidence remains limited. Only a small number of clinical trials have been completed to date, yet they provide important early insights into the translational relevance of preclinical findings. The following section reviews the outcomes of these initial trials.

In one of the first published studies, researchers investigated the effects of postbiotics on muscle function in adults and older adults using Urolithin A (UA). The data indicated significant improvements in leg muscle strength assessed by an isokinetic Biodex dynamometer, as well as aerobic endurance and overall physical performance, including a significant increase in walking distance during a 6 min walk test [88]. Similar findings were reported by Lee et al., who compared a probiotic form of *Lacticaseibacillus paracasei* PS23 (L-PS23) with its heat-treated postbiotic form (HT-PS23) [89]. In this study, lower limb muscle strength and endurance improved, as assessed by the 30 s chair stand and 2.44 m Timed Up-and-Go tests at weeks 6 and 12. While both groups exhibited positive outcomes after 12 weeks, improved performance was already observed in the postbiotic group at week 6, specifically in the 30 s chair stand test [89]. Notably, in both studies, upper limb grip strength remained unchanged, and no statistically significant changes in muscle or fat mass were detected [88,89].

In contrast, Cheng et al. reported that supplementation with heat-killed TWK10 cells improved grip strength in both the left and right hands, and significantly increased muscle mass in the subjects [90].

Beyond functional outcomes, both UA and HT-PS23 interventions were associated with reductions in proinflammatory markers. The former reduced CRP, interferon gamma (IFN-γ), IL-1β, and TNF-α [88], while the latter decreased IL-6 and increased anti-inflammatory IL-10 [89]. Additionally, in the HT-PS23 study, supplementation was associated with increased testosterone levels compared to placebo, suggesting a potential endocrine contribution to the observed effects [89].

In a separate randomized, double-blind, placebo-controlled trial, Kang et al. demonstrated that 12-week supplementation with a pasteurized *Akkermansia muciniphila*-derived postbiotic (HB05P) improved lower limb muscle strength in individuals aged 60 years and older, particularly in knee extensor muscles, as assessed by isokinetic dynamometry (Biodex System 3 Pro) [91]. These functional improvements were accompanied by increased circulating follistatin levels, a key antagonist of myostatin, suggesting modulation of pathways involved in muscle growth [91].

Published studies indicate that postbiotics yield superior outcomes compared to probiotic supplementation. HT-PS23 produced more favourable results in elderly individuals than the probiotic form of L-PS23, including improved performance in the chair stand test. The postbiotic formulation was also more effective in increasing testosterone levels than L-PS23. Furthermore, only HT-PS23 resulted in a significant increase in IGFBP-3 levels compared to the placebo group. These biomarkers are significant, as they reflect processes associated with muscle synthesis [89]. Evidence also suggests that treatment with heat-killed TWK10 in non-athletic adults yields effects comparable to those achieved with probiotic doses ten times higher [90]. These findings indicate that such preparations enhance physical performance and support muscle condition, which typically declines with age [89,90].

Another focus of postbiotic interventions is mitophagy, which refers to the selective removal of damaged or unnecessary mitochondria, coupled with the regeneration of mitochondrial populations [88,92]. Both aging and disease are associated with impaired mitophagy and reduced mitochondrial biogenesis, contributing to declines in cellular energy metabolism [93]. In this context, the study investigating UA supplementation at a dose of 500 mg reported significant activation of mitochondrial genes, suggesting stimulation of anabolic processes at the mRNA level [88]. These findings suggest that postbiotics may support mitochondrial quality control and energy metabolism by modulating pathways involved in mitophagy and mitochondrial biogenesis.

Clinical trials discussed above show that postbiotic supplementation may enhance muscle strength or function, endurance, and general physical performance, as well as counteract impaired mitophagy. Postbiotic therapy reinforces the human organism by reducing inflammation and enhancing signals that promote muscle growth. However, it is crucial to acknowledge the heterogeneity in the current literature, as some studies report isolated improvements in functional strength without accompanying increases in muscle mass, whereas others demonstrate concurrent gains in both parameters. Research also indicates that postbiotics might be more beneficial than probiotics in preventing age-related muscle loss.

## 6. Future Directions

Despite promising findings from preclinical studies indicating that postbiotic supplementation may beneficially modulate muscle atrophy and sarcopenia in animal models, these investigations represent only an initial step toward therapeutic application. Importantly, such studies suggest potential efficacy within controlled experimental settings rather than providing definitive evidence for clinical benefit in human populations. In contrast, the outcomes of currently available human studies remain limited and often inconclusive, hindering the ability to draw robust conclusions regarding the strength and consistency of postbiotic effects on human skeletal muscle physiology.

A clear translational gap persists, as there are very few well-designed, randomized controlled trials capable of providing meaningful clinical insight into the efficacy, optimal dosing, and mechanisms of postbiotic interventions in the context of muscle health. Future research should focus on bridging this gap by validating existing preclinical hypotheses through more extensive human cohorts. Table 4 summarizes ongoing clinical trials registered on ClinicalTrials.gov and the ISRCTN registry (accessed on 20 December 2025) that investigate the relationship between postbiotics and skeletal muscle outcomes [94,95,96,97].

Given the biological complexity of the gut–muscle axis and the diversity of postbiotic compounds, further rigorously designed clinical studies are required to validate existing hypotheses and support the translation of postbiotics into evidence-based strategies for the prevention or treatment of muscle loss.

## 7. Search Strategy

A comprehensive literature search was conducted using PubMed, Google Scholar, and Scopus databases. In addition to published studies, ongoing and registered clinical trials were identified through a screening process of the publicly available registries, including ClinicalTrials.gov and the ISRCTN Registry (www.isrctn.com) (accessed on 20 December 2025). Search queries were constructed using combinations of Medical Subject Headings (MeSH) and free-text phrases/keywords, including “postbiotic”, “heat-killed”, “sarcopenia”, “skeletal muscle”, “muscle strength”, “gut–muscle axis”, “microbiota”, “microbiome”, “dysbiosis”, “aging”, and “older”. The search prioritized studies published since 2020, with selective inclusion of relevant earlier publications from 2010 to 2019. All tables and figures were prepared by the authors through synthesis of the reviewed literature and extracted data. Figures were developed as original schematics using Sketchbook, GoodNotes 6, and diagrams.net (draw.io; accessed 6 January 2026).

## 8. Conclusions

This review highlights the emerging role of the gut-muscle axis as a critical regulator of skeletal muscle physiology and underscores the potential of postbiotics as a novel therapeutic strategy for counteracting age-related muscle deterioration. The synthesis of current evidence indicates that postbiotics, defined as preparations of inanimate microorganisms and their components, may represent a promising alternative to probiotics, with potential advantages in terms of stability, safety, and dosing consistency.

Amidst the rapid expansion of the aging population, with the number of older persons predicted to soon approach 2 billion, this research is critical for doctors, geriatricians, and scientists seeking advances in the battle against mobility impairment. By exploring the gut–muscle axis, we discuss how postbiotic supplementation could serve as a supportive strategy to mitigate muscle wasting, as current studies suggest that they can modulate pathways involved in slowing atrophy and preserving muscle strength.

Preclinical models suggest that postbiotic supplementation may attenuate muscle atrophy and enhance functional outcomes, such as grip strength and endurance capacity. These beneficial effects appear to be mediated through a synergistic interplay of molecular mechanisms, including activation of anabolic mTOR signalling, inhibition of ubiquitin-proteasome-dependent catabolism, and modulation of systemic inflammation and oxidative stress. Furthermore, postbiotics may contribute to intestinal homeostasis by strengthening the epithelial barrier and regulating distinct immune pathways, potentially mitigating dysbiosis often associated with sarcopenia.

However, the translation of these promising preclinical findings into clinical practice remains at an early stage. While initial human trials suggest benefits for physical performance and inflammatory markers, the evidence regarding muscle mass accretion in humans is currently less consistent, with some interventions improving function without significant morphological changes. A significant translational gap persists due to the limited number of large-scale randomized controlled trials and the high heterogeneity of postbiotic compounds tested to date.

Consequently, while the hypotheses outlined here provide a promising framework, postbiotics should currently be regarded as a potential, rather than an established, approach to supporting muscle health in aging populations. Their validation will require rigorous and systematic investigation. Future research should prioritize well-designed randomized controlled trials, alongside efforts to standardize postbiotic formulations, define optimal dosing strategies, and clarify the specific effects of different postbiotic strains on human muscle biology.

## Figures and Tables

**Table 1:** The impact of gut bacteria on skeletal muscle and their mechanisms of action [38,40,41].

Bacterial Species/Taxa	Effect on Skeletal Muscle	Mechanism of Action	References
*Lactobacillus johnsonii*	↑ muscle strength	Upregulation of IGF-1 and follistatin; stimulation of muscle growth pathways	[38]
*Limosilactobacillus reuteri*	↑ muscle strength	Increased IGF-1 and follistatin signalling; enhanced muscle performance	[38]
*Faecalibacterium prausnitzii*	Preservation of muscle mass	SCFA production; enhanced energy metabolism and muscle regeneration	[40]
*Roseburia inulinivorans*	Support of muscle metabolism	SCFA production; enhanced energy metabolism and muscle regeneration	[40]
*Lactobacillus* spp. (BSH-active)	Maintenance of muscle mass	Bile salt hydrolase activity → secondary bile acid production → FXR–FGF15/19 signalling supporting protein synthesis	[41]
*Bifidobacterium* spp. (BSH-active)	Protection against atrophy	Regulation of bile acid metabolism and FXR signalling; improved muscle anabolic pathways	[41]

Abbreviations: IGF-1—Insulin-like Growth Factor 1; SCFAs—Short-chain fatty acids; BSH—bile salt hydrolases; FXR—farnesoid X receptor; FGF15/19—fibroblast growth factor 15/19.

**Table 2:** Classification of Postbiotics into Bacterial Components and Metabolites [50].

Postbiotic Group	Postbiotic Examples
Bacterial components	LPS, muropeptides from peptidoglycan, flagellin, LTA, and DNA-containing EVs.
Bacterial metabolites	SCFAs, secondary bile acids, trimethylamine oxide, D-lactate, glycerol, succinate, ethanolamine, and ethanol

Abbreviations: LPS—lipopolysaccharides, LTA—lipoteichoic acids, EVs—extracellular vesicles, SCFAs—Short-Chain Fatty Acids.

**Table 3:** Overview of postbiotic mechanisms of action with representative examples related to muscle health [32,50,54,57,59,60,61,62,63,64,65,66,67,68,69].

Functional Domain	Postbiotic Components	Primary Targets/Pathways	Key Effects	Representative Examples	References
Modulation of resident microbiota	Organic acids, SCFAs, EPS, bacteriocins, cell-free supernatants	Pathogenic and commensal bacteria; QS disruption; microbial cross-feeding	Suppression of pathogens, promotion of beneficial bacteria, increased SCFA production, inhibition of biofilm formation	EPS from *Lactobacillus paracasei* enhances propionate and butyrate production [60]; bacteriocins from *L. acidophilus* KS40 suppress urogenital pathogens [61]; *L. rhamnosus* XN2 disrupts QS showing bactericidal activity [69]	[32,54,57,60,61,69]
Epithelial barrier integrity	EPS, p40 and p75 proteins, fatty acids (oleic acid, palmitic acid)	Tight-junction proteins (ZO-1, occludin, claudins); STAT3; PKC/MAPK; EGFR	Enhanced intestinal barrier function, reduced permeability, protection against epithelial apoptosis, decreased inflammation	EPS from *L. plantarum* upregulates ZO-1 and occludin via STAT3 [62]; p40/p75 preserve tight junctions [32]; fatty acids alleviate colitis and enhance antifungal defense [63]	[32,59,62,63]
Regulation of immune responses	LTA, peptidoglycans, pili, flagella, CpG-DNA, SCFAs, indoles, EVs, succinate	TLR2/6, TLR2/5, TLR9, NOD2, EGFR, cGAS/STING, SUCNR1	Immune maturation, balanced Th17/Treg responses, reduced apoptosis, anti-inflammatory cytokine profiles	Postbiotics from *L. helveticus* KLDS 1.8701 modulate Th17/Treg balance [64]; EVs activate cGAS/STING [50]; succinate engages SUCNR1 [50]	[32,50,54,64,65]
Regulation of metabolic pathways	SCFAs (acetate, propionate, butyrate)	GPR43/GPR41, AMPK, PPAR-γ, GPR41/43, ERK/MAPK	Improved energy metabolism, enhanced insulin sensitivity, increased fatty acid oxidation, suppression of gluconeogenesis, stimulation of muscle regeneration	Butyrate promotes C2C12 myoblast proliferation via ERK/MAPK pathway activation [67]; SCFAs stimulate GLP-1 and PYY secretion [66]	[50,66,67]
Neuroendocrine and gut–brain signalling	Neuroactive metabolites (GABA, serotonin, dopamine, histamine, acetylcholine), BDNF	Enteric nervous system, vagal pathways, gut–brain axis	Modulation of neurotransmitter balance, vagal signalling, neurochemical homeostasis	Postbiotics from *L. plantarum* alter levels of 5-HT, GABA, dopamine, acetylcholine, BDNF, NPY in mice [68]	[32,68]

Abbreviations: SCFAs—Short-chain fatty acids; EPS—exopolysaccharides; QS—quorum sensing; ZO-1—Zonula Occludens-1; STAT3—Signal Transducer and Activator of Transcription 3; PKC/MAPK—Protein Kinase C/Mitogen-Activated Protein Kinase; EGFR—Epidermal Growth Factor Receptor; LTA—Lipoteichoic acid; CpG-DNA—Cytosine-phosphate-Guanine DNA; EVs—extracellular vesicles; TLR—Toll-like receptor; NOD—Nucleotide-binding Oligomerization Domain; cGAS/STING—cyclic GMP-AMP synthase/Stimulator of Interferon Genes; SUCNR1—Succinate receptor 1; AMPK—AMP-activated protein kinase; PPAR-γ—Peroxisome Proliferator-Activated Receptor Gamma; GPR—G Protein-Coupled Receptors; ERK/MAPK—Extracellular signal-regulated kinase/Mitogen-activated protein kinase; GLP-1—Glucagon-like Peptide-1; PYY—Peptide YY; GABA—gamma-Aminobutyric acid; BDNF—Brain-Derived Neurotrophic Factor; 5-HT—5-hydroxytryptamine; NPY—Neuropeptide Y.

**Table 4:** Ongoing clinical trials investigating the effects of postbiotic supplementation on muscle health, registered in ClinicalTrials.gov and the ISRCTN registry [94,95,96,97].

ClinicalTrials, GOV ID/ISRCTN ID, Research Status	Research Area and Intervention	Study Population	Purpose of the Study	References
NCT06230302; Enrolling by invitation	Gut-muscle axis: postbiotics from kefir whey	Elderly people over 40 years of age	Evaluation of effectiveness in improving grip strength, safety, impact on biomarkers such as adipokines, myokines, lipids and cytokines, and evaluation of modification of the gut microbiome	[94]
NCT06428656; Recruiting	Sports performance and regeneration: heat-killed *Lactobacillus plantarum* L-137, Immuno-LP20TM	Physically active people from 18 to 45 years of age	The effect of postbiotics on post-exercise inflammation, regeneration after oxidative stress, body performance after intense eccentric exercise and the composition of the microbiota	[95]
ISRCTN47159002; Ongoing	Muscle health: Urolithin A (Mitopure^®^)	Healthy people aged 40–65	Investigation of the effect of Urolithin A on muscle strength and performance in healthy middle-aged adults	[96]
ISRCTN20052152; Completed	Regeneration and atrophy: protein drink with Urolithin A	Healthy men aged 18–30, immobilized with a knee brace	Evaluation of the effectiveness of nutritional intervention to prevent muscle atrophy induced by inactivity while wearing a brace	[97]

## Data Availability

This study is based on publicly available data retrieved from databases including PubMed, Scopus, Google Scholar, and publicly available registries, including ClinicalTrials.gov and the ISRCTN Registry. No new data were generated or analyzed by the authors.
